# Lumbar vertebral pattern variation in the common opossum (*Didelphis marsupialis* Linnaeus, 1758): implication on lumbar nerve distribution

**DOI:** 10.1080/23144599.2022.2163561

**Published:** 2023-01-05

**Authors:** Andrés Sepúlveda-Vásquez, Lynda Tamayo-Arango

**Affiliations:** Grupo de Investigación CIBAV, Escuela de Medicina Veterinaria, Facultad de Ciencias Agrarias, Universidad de Antioquia UdeA, Medellín, Colombia

**Keywords:** Lumbosacral plexus, *didelphis marsupialis*, neuroanatomy, wild animals

## Abstract

The lumbar nerve distribution can differ depending on vertebral count variations among individuals of the same species. The variation in the lumbar vertebra formula and the lumbar nerve distribution in twenty adult common opossums (eight female and twelve males) was studied. Radiographs were taken to confirm vertebral identification and count. Two vertebral patterns were recognized: three specimens presented five lumbar vertebrae (5VP) and seventeen individuals presented six lumbar vertebrae (6VP). All the 6VP specimens had the same innervation pattern; however, the 5PV had three different innervation patterns (5PVa, 5VPB, and 5PVc). 5VPa and 6VP differed only in the origin of the lateral femoral cutaneous nerve (L2-L3 and L3, respectively). The differences among 5PVa, 5PVb, and 5VPc were seen in the iliohypogastric nerve, which was formed by L1 in 5VPa and 5VPb, and T13 in 5VPc. The ilioinguinal nerve was formed by L1-L2 in 5VPa and 5VPb, while it was formed by T13-L1 in 5VPc. The genitofemoral nerve was formed by L2-L3 in 5VPa, L2 in 5VPb, and L1-L2 in 5VPc. The cutaneous femoris lateralis was formed by L2-L3 in 5VPa and 5VPc, while it is formed only by L2 in 5VPb. The femoral and obturator nerves were formed by L3-L4 in 5VPa, and L2-L3 in 5VPb and 5VPc. The lumbosacral trunk originated from L4-L5-S1 in 5VP and L5-L6-S1 in 6VP. The data provided in this study may help understand the relationship between the spine and lumbosacral plexus variations and may find application in veterinary spine surgery.

## 1. Introduction

The common opossum (*Didelphis marsupialis)* is one of the six species of the genus *Didelphis*. Opossums are nocturnal, nomadic, and highly opportunistic omnivorous marsupials that habit only in America [1–5]. The members of the genus *Didelphis* are very adaptable due to their generalist habits [6–8]. *D. marsupialis* prefers tropical, humid, broad-leaved forests due to their survival strategy that consists of high production and fast development of the young [2,9]. Therefore, opossums seem like a good option for animal biomodel.

The somites determine the segmental position of spinal nerves. Every spinal nerve will develop in the ventral half of each somite. Both somites and spinal nerves originate depending on their position along the axial series. Then somites will form the neck, thoracolumbar, and sacral structures. The number of segments that form each of these parts and their position along the axial series differs between different species and individuals of the same species, influencing the spinal nerve count and distribution [10].

The vertebral formula is variable in several mammal species. Nearly all mammals have seven cervical vertebrae regardless of the neck length; this is the case with whales and giraffes, for example. Also, almost all mammals have 19 thoracolumbar vertebrae (13 thoracic and 6 lumbar); nevertheless, post-cervical vertebral formula changes between orders and families. Carnivores, for example, have 20 thoracolumbar vertebrae [11,12].

The white-eared opossum (*Didelphis albiventris*) presents three different vertebrae formulas, C7/T13/L6/S2 is the usual formula, and the lumbar vertebrae count could vary between L5, L6, and L7. This finding suggests that *the Didelphis* genus could present variations in the vertebral formula; nevertheless, lumbosacral plexus has not been reported in this species, and the variations in the lumbar nerve’s origin and distribution are unknown [13].

The lumbosacral plexus has only been described in two of the six species of *Didelphis, D. aurita* [14] and *D. virginiana* [15]. However, no variations in the vertebral formula were reported in these studies. It is necessary to describe the lumbosacral plexus in other opossum species to identify if these differences are also present. This study aimed to investigate if there is a variation in the lumbar vertebral formula and its influence on the lumbar nerve distribution in the common opossum (*D. marsupialis*). The data provided in this study may help understand the relationship between the spine and lumbosacral plexus variations and may find application in veterinary spine surgery.

## 2. Materials and methods

### 2.1. Ethical statement

This study was approved by the Committee on Ethics in Animal Research of the Universidad de Antioquia, Act 134 of August 2020.

### 2.2. Specimen source

Twenty adult opossums’ cadavers (eight females and twelve males) were donated to the Laboratory of Animal Anatomy of the Universidad de Antioquia by the environmental authority and persons that found them dead in the field.

### 2.3. Fixation method

Eighteen cadavers were conserved using a solution based on ethanol, benzalkonium chloride, and propylene glycol via the common carotid artery [16]. Two cadavers were injected with red-coloured latex via the common carotid artery and blue-coloured latex via the superficial jugular vein and then preserved by intramuscular injection and immersion into the fixative solution mentioned above.

### 2.4. Radiological study

Lateral radiographs were taken to the thoracic, lumbar as well as sacral vertebrae to verify vertebral identification and count.

### 2.5. Dissection method

A conventional bilateral dissection was performed to identify and expose the muscles, nerve roots, and branches of the lumbar region and the pelvic limbs. Vascular structures were removed for better visualization of the nerves. A ventral approach was performed to expose the lumbosacral plexus, and the abdominal viscera and hypaxial musculature were removed for better exposure of the vertebral bodies and the spinal nerves. Vertebrae were carefully counted, and corresponding pairs of spinal nerves were identified. Then, a lateral approach was performed at the proximal level of the femoral region to identify the sciatic nerve and dissect all the lumbosacral plexus branches following it proximally and distally. The lumbosacral trunk and the sciatic nerve were further dissected to differentiate the spinal origin of each of all branches. The anatomical description followed the Nomina Anatomica Veterinaria [17].

### 2.6. Photography

Digital photographs were taken with an iPhone 7 plus (12 megapixels) and a Nikon d5500 + Nikon AF-S DX NIKKOR 18–55 mm, then edited in the iPhone 7 Plus using a free version of Lightroom and Photoshop fix.

## 3. Results

**Figure 1.**
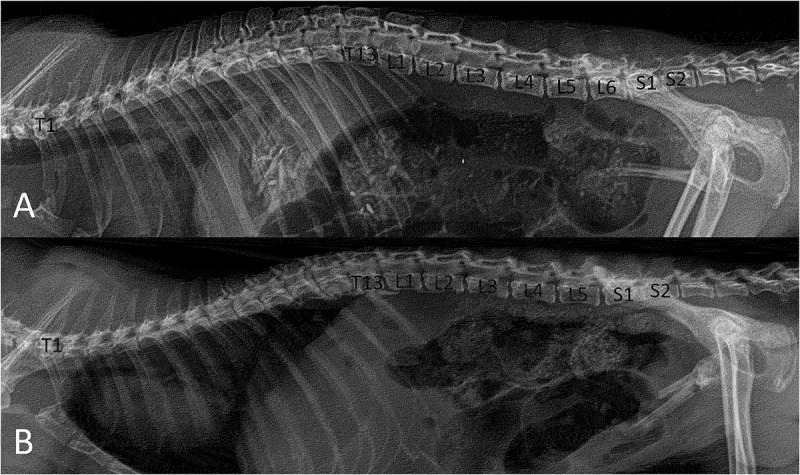
Lateral radiograph of the lumbosacral spine in Didelphis marsupialis 5VP (A) and 6VP (B). T13, thirteenth thoracic vertebrae; L1, first lumbar vertebrae; L2, second lumbar vertebrae; L3, third lumbar vertebrae; L4, fourth lumbar vertebrae; L5, fifth lumbar vertebrae; L6, sixth lumbar vertebrae; S1, first sacral vertebrae; S2, second sacral vertebrae.

**Figure 2.**
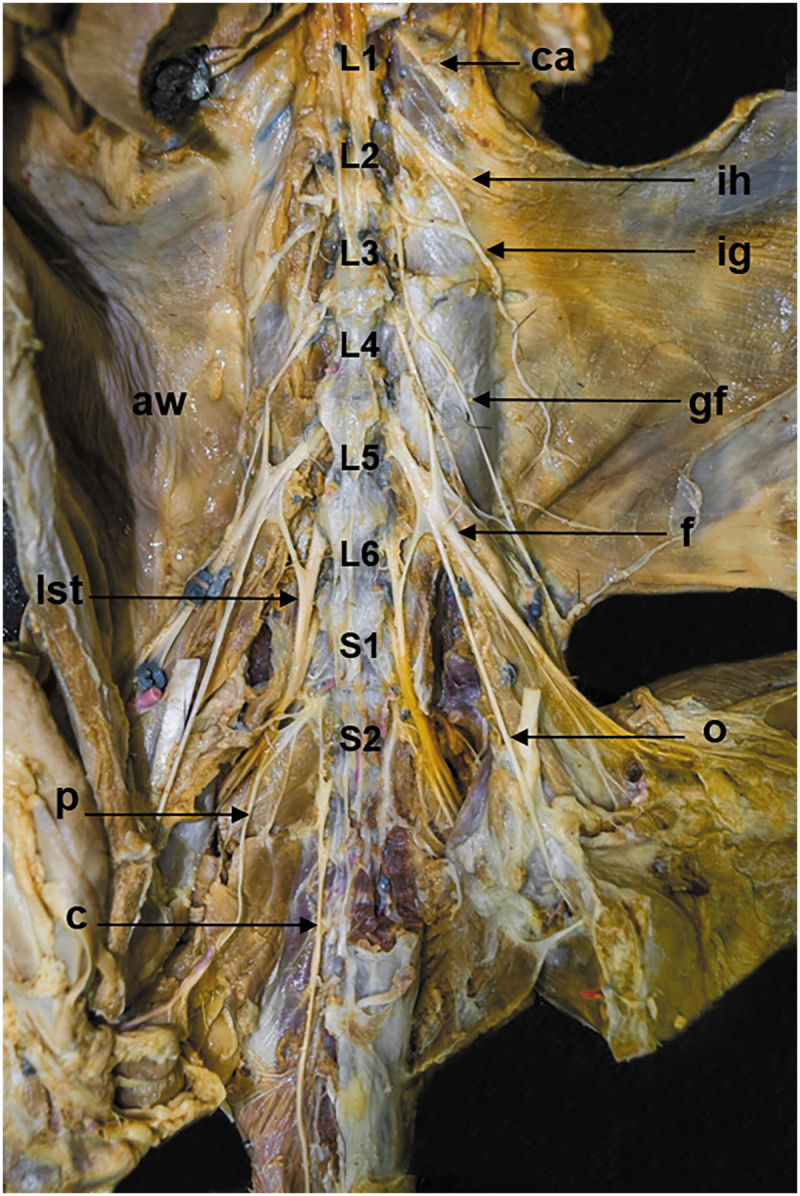
Ventral view of the lumbosacral plexus in a common opossum with 6PV. *aw*, abdominal wall; *c*, caudal nerve; *ca*, costoabdominal nerve; *f*, femoral nerve; *gf*, genitofemoral nerve; *ih*, iliohypogastric nerve; *ig*, ilioinguinal nerve; *L1*, first lumbar vertebrae (lv); *L2*, second lv; *L3*, third lv; *L4*, fourth lv; *L5*, fifth lv; *L6*, sixth lv; *lst*, lumbosacral trunk; *S1*, first sacral vertebrae (sv); *S2*, second sv; *o*, obturator nerve; *p*, pudendal nerve.

**Figure 3.**
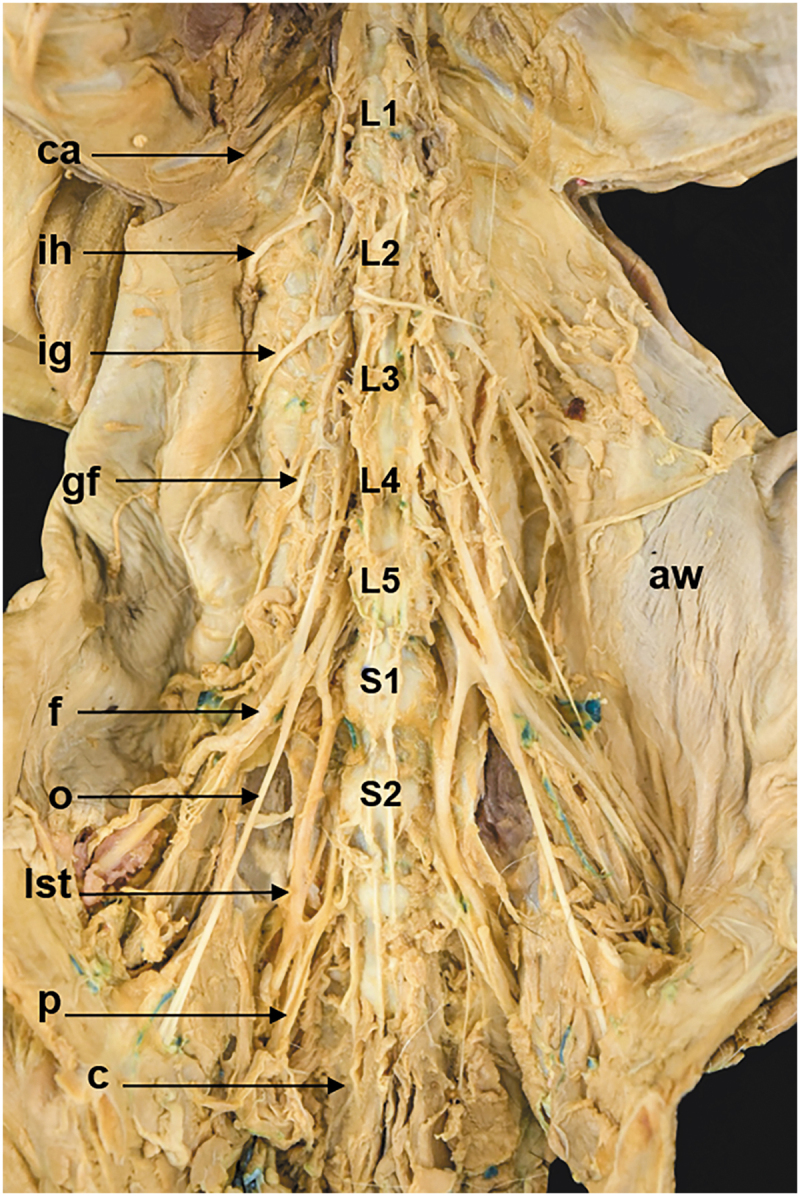
Ventral view of the lumbosacral plexus roots in a common opossum with 5PVa. *aw*, abdominal wall; *c*, caudal nerve; *ca*, costoabdominal nerve; *f*, femoral nerve; *gf*, genitofemoral nerve; *ih*, iliohypogastric nerve; *ig*, ilioinguinal nerve; *L1*, first lumbar vertebrae (lv); *L2*, second lv; *L3*, third lv; *L4*, fourth lv; *L5*, fifth lv; *lst*, lumbosacral trunk; *s1*, first sacral nerve; *o*, obturator nerve; *p*, pudendal nerve.

**Figure 4.**
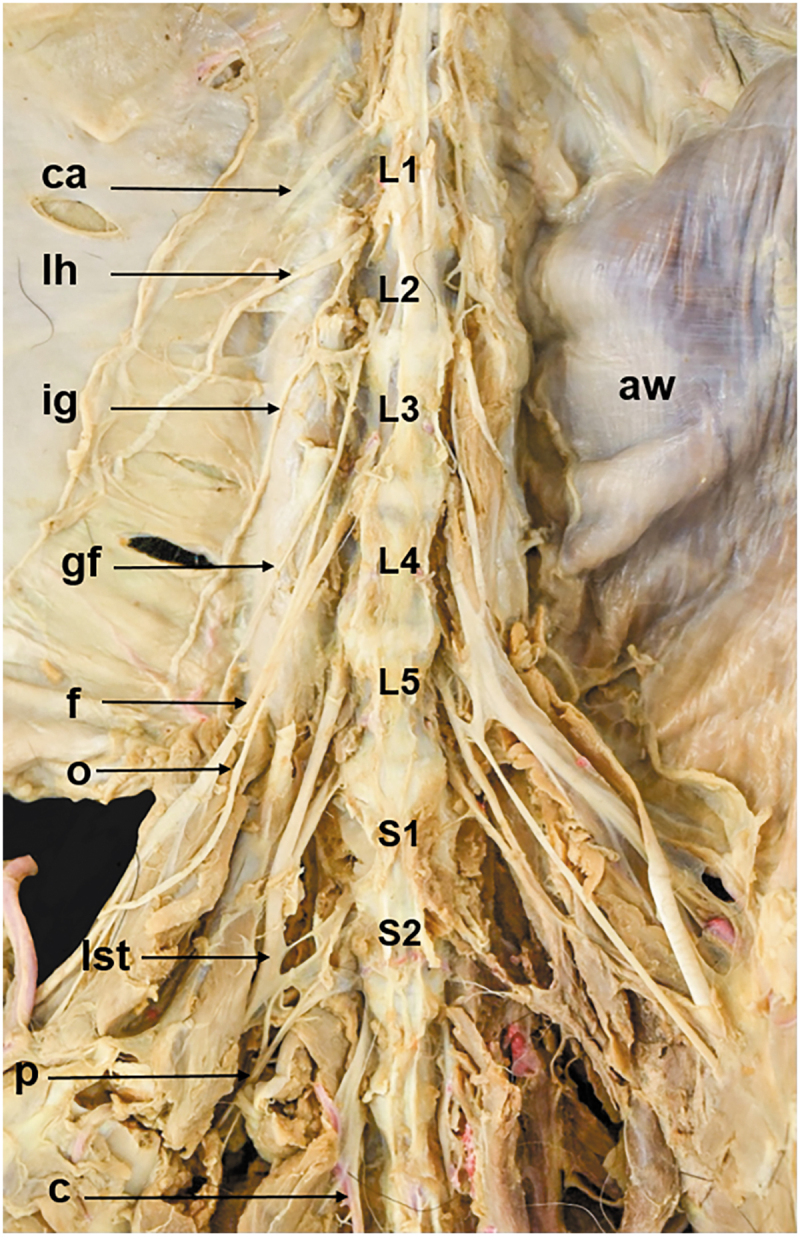
Ventral view of the lumbosacral plexus roots in a common opossum with 5PVb. *aw*, abdominal wall; *c*, caudal nerve; *ca*, costoabdominal nerve; *f*, femoral nerve; *gf*, genitofemoral nerve; *ih*, iliohypogastric nerve; *ig*, ilioinguinal nerve; *L1*, first lumbar vertebrae (lv); *L2*, second lv; *L3*, third lv; *L4*, fourth lv; *L5*, fifth lv; *lst*, lumbosacral trunk; *s1*, first sacral nerve; *o*, obturator nerve; *p*, pudendal nerve.

**Figure 5.**
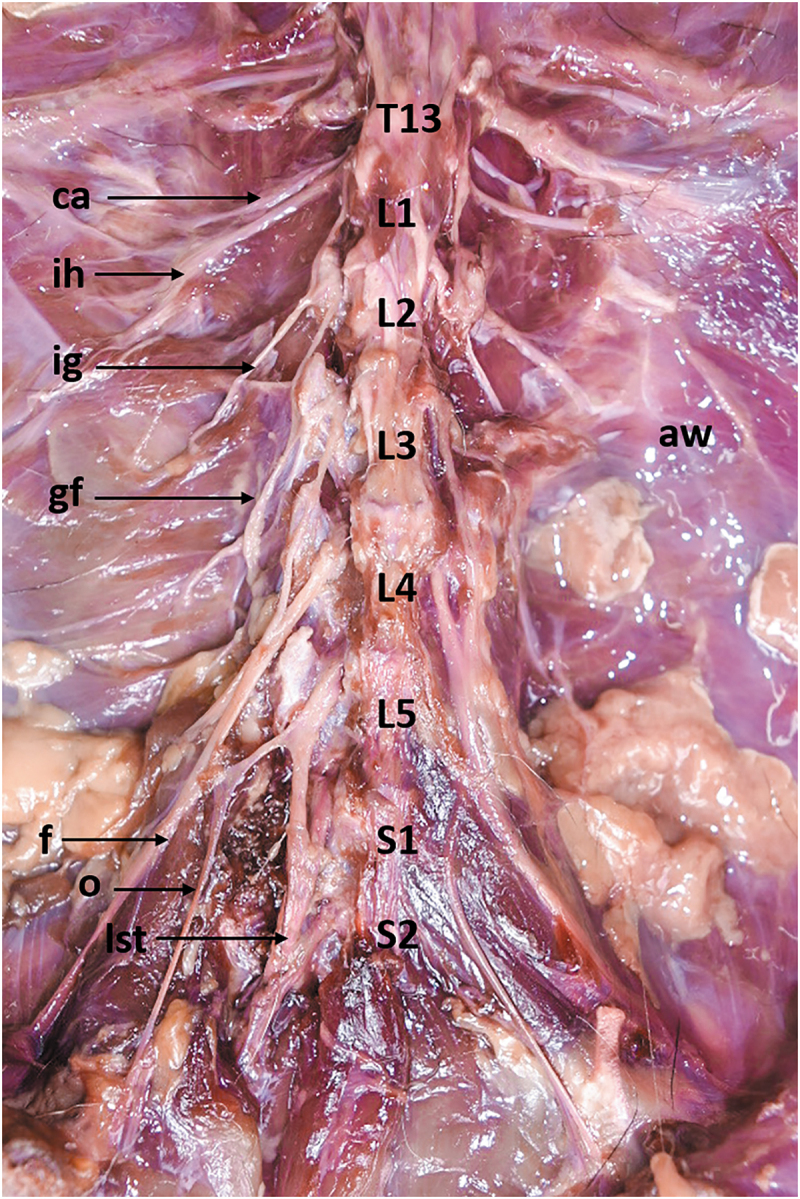
Ventral view of the lumbosacral plexus roots in a common opossum with 5PVc. *aw*, abdominal wall; *c*, costoabdominal nerve; *f*, femoral nerve; *gf*, genitofemoral nerve; *ih*, iliohypogastric nerve; *ig*, ilioinguinal nerve; T13, last thoracic vertebrae; *L1*, first lumbar vertebrae (lv); *L2*, second lv; *L3*, third lv; *L4*, fourth lv; *L5*, fifth lv; *lst*, lumbosacral trunk; *s1*, first sacral nerve; *o*, obturator nerve.

Two vertebral patterns were recognized: three specimens (15%, one female and two males) presented five lumbar vertebrae (5VP), and seventeen specimens (85%, seven females and ten males) presented six lumbar vertebrae (6VP) (Figure 1, Table 1). These vertebral patterns implied the origin of the spinal nerves of the lumbosacral plexus. The 6VP specimens had the same innervation pattern (Figure 2); however, the 5PV had three different innervation patterns (5PVa,5PVb, and 5PVc) (Figures 3, 4, and 5).

**Table 1.** Nerve distribution patterns by sex (*n* and percentages).

Nerve distribution pattern	6VP	5VPa	5VPb	5VPc
Male	10 (50%)	1 (5%)	0 (0%)	1 (5%)
Female	7 (35%)	0 (0%)	1 (5%)	0 (0%)
Total	17 (85%)	1 (5%)	1 (5%)	1 (5%)

One specimen (5%) presented the 5VPa pattern, where the iliohypogastric nerve was formed by L1, the ilioinguinal nerve was formed by L1-L2, the genitofemoral was formed by L2-L3, the femoral and obturator nerves were formed by L3-L4, and the lumbosacral trunk was formed by L4-L5-S1. One specimen (5%) presented the 5VPb pattern, where the iliohypogastric nerve was formed by L1, the ilioinguinal nerve was formed by L1-L2, the genitofemoral was formed by L2, the femoral and obturator nerves were formed by L2-L3, and the lumbosacral trunk was formed by L4-L5-S1. One specimen (5%) presented the 5VPc pattern, where the iliohypogastric nerve was formed by T13, the ilioinguinal nerve was formed by T13-L1, the genitofemoral was formed by L1-L2, the femoral and obturator nerves were formed by L2-L3, and the lumbosacral trunk was formed by L4-L5-S1.

The lumbosacral plexus (*plexus lumbosacralis*) was formed by the ventral branches of the first lumbar to the first sacral nerves (L1-S2) (*rami ventralis, nervi lumbales L1-L5 or L6, nervi sacrales S1-S2*) which originate several nerves that innervate the abdominal wall, the pelvic limb, the pelvic cavity viscera, and the tail. The spinal origin and distribution of the lumbosacral plexus nerves are summarized in Table 2.

**Table 2.** Spinal origin and distribution of the lumbosacral plexus nerves in the common opossum (*Didelphis marsupialis).*

Nerve	Spinal origin				Motor innervation (muscle)	Sensory innervation
	5VPa	5VPb	5VPc	6VP		
Iliohypogastric	L1	L1	T13	L1	Abdominal wall (1)	Abdominal wall (1)
Ilioinguinal	L1, L2	L1, L2	T13, L1	L1, L2	Abdominal wall (1)	Abdominal wall (1)
Genitofemoral	L2, L3	L2	L1, L2	L2, L3	Abdominal wall (1), cremaster (1)	Abdominal wall (1), spermatic cord, and scrotum
lateral femoral cutaneous	L2, L3	L2	L2, L3	L3	None	Femoral cranial lateral region
Femoral	L3, L4	L2, L3	L2, L3	L3, L4	Psoas minor (3), quadriceps femoris (4): vastus lateral (1), vastus medial (1), vastus intermediate (1), rectus femoris (1). Saphenous nerve: sartorius (1)	Saphenous: medial aspect of the pelvic limb and first digit
Obturator	L3, L4	L2, L3	L2, L3	L3, L4	Pectineal (1), adductor (1), gracilis (1), internal obturator (1), external obturator (1)	None
Lumbosacral trunk	L4, L5, S1	L4, L5, S1	L4, L5, S1	L5, L6, S1	Superficial gluteal (1)	None
Sciatic	L4, L5	L4, L5	L4, L5	L5, L6	Biceps femoris (2), semitendinosus (4), semimembranosus (1), caudal abductor of crus (1), cranial gemellus (1), caudal gemellus (1), quadratus femoris (1)	None
Branch from sciatic for the ischiotibial muscles	L4, L5	L4, L5	L4, L5	L5, L6		None
Cranial gluteal	L4	L4	L4	L5	Middle gluteal (1), Superficial gluteal (1), deep gluteal (2)	
Caudal gluteal	L4, L5	L4, L5	L4, L5	L5, L6	Superficial gluteal (1), gluteofemoral (1), piriform (1)	None
Caudal femoral cutaneous	S1	S1	S1	S1	None	Femoral caudal lateral region
Tibial	L3, L4, L5	L3, L4, L5	L3, L4, L5	L4, L5, L6	Triceps surae (1), plantaris (2), medial digital flexor & short digital flexor (1), long digital flexor muscle of the first digit (1), caudal tibial (2), popliteal (1). Caudal sural cutaneous nerve: gastrocnemius medial head (1), semitendinosus (1). Lateral plantar nerve: short digital flexor of the first digit (2), interosseous (3)	Caudal sural cutaneous nerve: lateral region of the foot (1). Medial plantar nerve: I to IV digits and their pads (5). Lateral plantar nerve: digit V, IV and their pads (3).
Common fibular	L3, L4, L5	L3, L4, L5	L3, L4, L5	L4, L5, L6	Gastrocnemius lateral head (1) Biceps femoris (1) Deep fibular nerve: fibularis longus (2), fibularis brevis (1), lateral digital extensor (1) Short digital extensor 1), long digital extensor (1), cranial tibial (2), long extensor of the first digit (1), short extensor of the first digit (1) lumbrical (2)	dorsal region of the foot, digits ll to V. Superficial fibular nerve: dorsal region of the foot
Pudendal	L5, S1	L5, S1	L5, S1	L6, S1	External anal sphincter (1), hypaxial coccygeal musculature (1)	penis (1), urogenital canal (1), bulbourethral glands (3), paranal sinus (1), perineal region (1)
Pelvic	S1	S1	S1	S1	None	uterus (1), prostate (1), urinary bladder (1)
Caudal	S1	S1	S1	S1	Hypaxial coccygeal musculature (1)	None

*In parentheses is the number of branches that innervate each structure.

### 3.1. Iliohypogastric nerve

The first lumbar nerve (L1) formed the iliohypogastric nerve (*N. iliohypogastricus*) in all specimens except for 5VPc, in which it was formed by T13. It provided innervation to the cranial part of the abdominal wall.

### 3.2. Ilioinguinal nerve

The ilioinguinal nerve (*N. ilioinguinalis*) was formed by L1 and L2 in all specimens except for 5VPc, in which it was formed by T13 and L1. It sent branches to the abdominal wall, quadratus lumborum muscle, and iliopsoas muscle.

### 3.3. Genitofemoral nerve

The genitofemoral nerve (*N. genitofemoralis*) ran in the spermatic cord and was formed by L2 and L3 in 5VPa, only by L2 in 5VPb, L1 and L2 in 5VPc, and by L2 and L3 in 6VP.

### 3.4. Lateral femoral cutaneous nerve

The lateral femoral cutaneous nerve (*N. cutaneus femoris lateralis*) was formed by L2 and L3 in 5VPa and 5VPc, only by L2 in 5VPb, and only by L3 in 6VP. The lateral femoral cutaneous nerve provided innervation to the caudal part of the abdominal wall and psoas minor muscle.

### 3.5. Femoral and obturator nerves

The femoral (*N. femoralis*) and obturator (*N. obturatorius*) nerves were formed by L3 and L4 in 5VPa, by L2 and L3 in 5VPb and 5VPc, and L3 and L4 in 6VP. The femoral and obturator nerves were observed in the medial view of the proximal portion of the femoral region. The femoral nerve ran between the quadrate lumbar muscle and iliopsoas muscle towards the femoral triangle to join the femoral artery and vein. At the same time, it provided branches to the psoas minor muscle. Once it was on the proximal region of the pelvic limb, it originated the saphenous nerve (*N. saphenus*). It then gave off four terminal branches for the innervation of the quadriceps femoris muscle, one branch for each head. The saphenous nerve innervated the sartorius muscle and ran distally along the medial surface of the pelvic limb, innervating the skin of this region, ending at the dorsal portion of the thumb. The obturator nerve ran towards the obturator foramen, innervating the external obturator muscle before going through it to innervate the internal obturator muscle and the adductor muscles of the thigh: The pectineal, the adductor, and the gracilis.

### 3.6. Lumbosacral trunk and cranial gluteal nerve

The lumbosacral trunk (*Truncus lumbosacralis*) was formed by L4, L5, and S1 in 5VPa, 5VPb and 5VPc, and from L5, L6, and S1 in 6VP. It was the thickest nerve of the plexus and provided a direct branch to the superficial gluteal muscle. Then, it originated the cranial gluteal nerve (*N. gluteus cranialis*) and continued as the sciatic nerve (*N. ischiadicus*). The cranial gluteal nerve ran over the greater sciatic incisura to provide motor innervation for the gluteal muscles, sending branches to the superficial gluteal muscle, the middle gluteal muscle, and the deep gluteal muscle.

### 3.7. Sciatic and caudal gluteal

**Figure 6.**
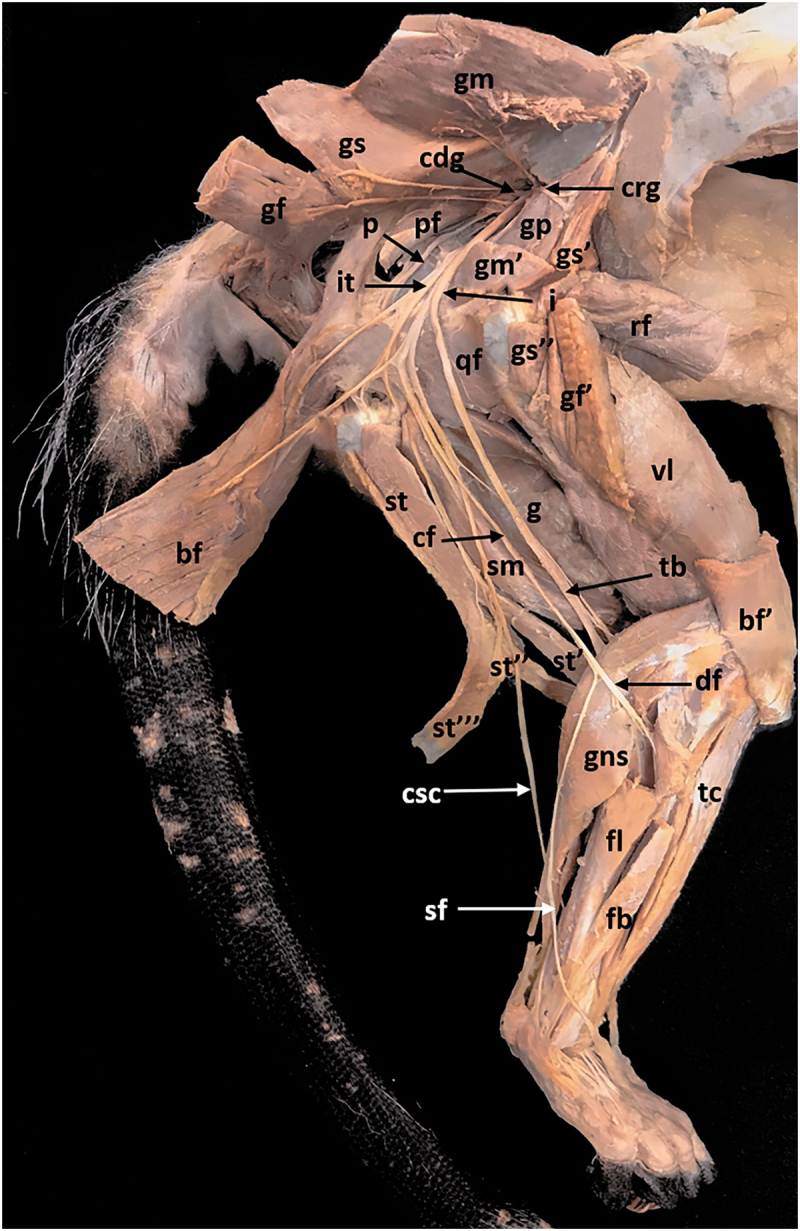
Lateral view of the pelvic limb. *bf*, biceps femoris muscle; *bf’*, biceps femoris muscle insertion; *cag*, caudal gluteal nerve; *cf*, common fibular nerve; *crg*, cranial gluteal nerve; *csc*, caudal sural cutaneous nerve; *df*, deep fibular nerve; *fb*, fibularis brevis muscle; *fl*, fibularis longus muscle; *g*, gracilis muscle; *gf*, gluteus femoris muscle; *gf’*, gluteus femoris muscle insertion; *gm*, gluteus medius muscle; *gm’*, gluteus medius muscle insertion; *gns*, gastrocnemius-soleus muscle; *gp*, gluteus profundus muscle; *gs*, gluteus superficialis muscle; *gs’*, gluteus superficialis muscle iliac insertion; *gs”*,gluteus superficialis muscle trochanteric insertion; *i*, Sciatic nerve; *it*, ischiotibial branch; *rf*, rectus femoris muscle; *p*, pudendal nerve; *pf*, piriformis muscle; *qf*, quadratus femoris muscle; *sf*, superficial fibular nerve; *sm*, semimembranosus muscle; *st*, semitendinosus muscle; *st’*, semitendinosus muscle cranial medial insertion; *st”*, semitendinosus muscle caudal medial insertion; *st”’*, semitendinosus muscle lateral insertion; *tb*, tibial nerve; *tc*, tibialis cranialis muscle; *vl*, vastus lateralis muscle.

The sciatic nerve provided a branch for the ischiotibial muscles, from which the caudal gluteal nerve (*N. gluteus caudalis*) was derived. The caudal gluteal nerve provided motor innervation to the piriformis muscle and the gluteofemoral muscle. After that, the branch mentioned above that innervated the ischiotibial muscles ran under the piriformis muscle and sent a branch to the caudal muscles of the hip (gemelli muscles and quadratus femoris muscle). Then continued along the femoral region caudally to the hip joint and sent a proximal branch to the biceps femoris muscle, the semitendinosus muscle, the semimembranosus muscle, and the caudal abductor of crus; and a distal branch to the biceps femoris muscle and terminal branches for each insertion of the semimembranosus muscle (two for the medial proximal insertion, one for the medial distal insertion and one for the lateral insertion). The sciatic nerve, after the branch for the ischiotibial muscles, ran over the piriformis muscle medially and caudally to the hip joint, and it was divided into the tibial nerve *(N. tibialis)* and the common fibular nerve (*N. fibularis communis*) (Figure 6).

### 3.8. Caudal sural cutaneous nerve

**Figure 7.**
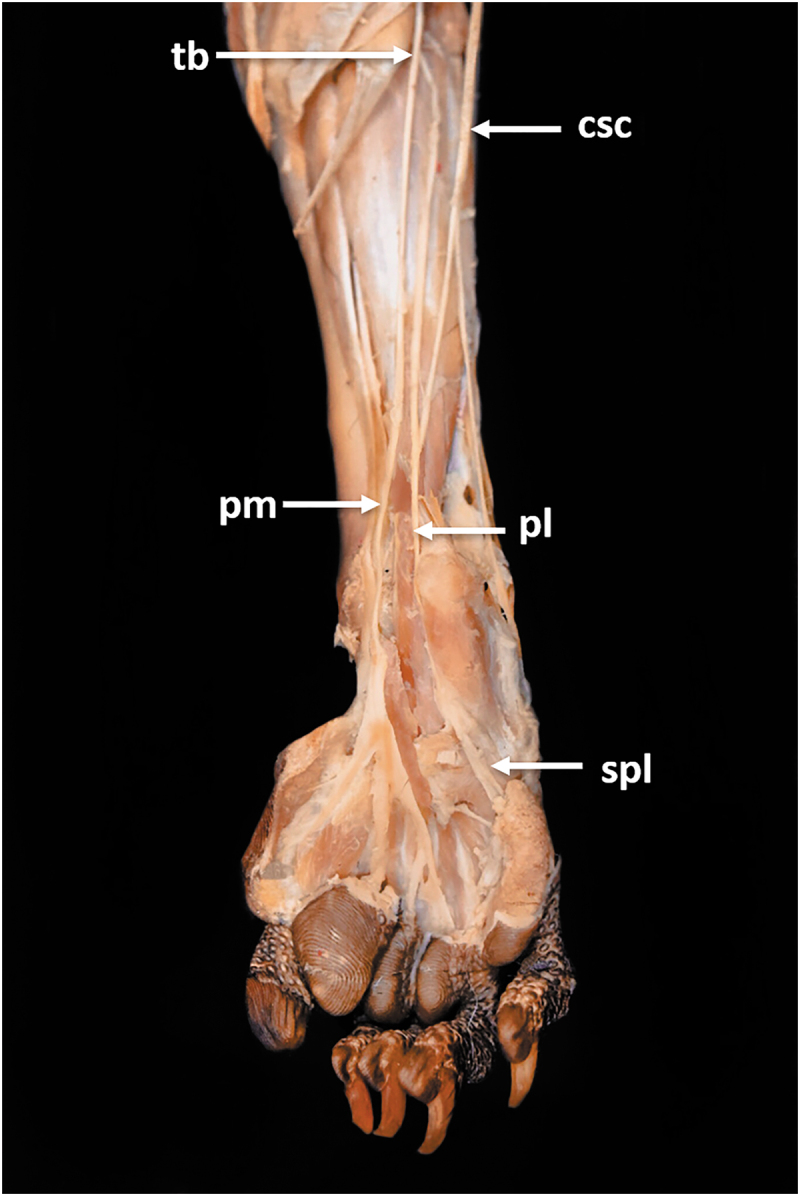
Plantar view of the foot. *csc*, caudal sural cutaneous nerve; *pl*, lateral plantar nerve; *pm*, medial plantar nerve; *spl*, lateral plantar nerve superficial branch; *tb*, tibial nerve.

The caudal sural cutaneous nerve (*N. cutaneus surae caudalis*) was derived from the tibial nerve. It provided innervation to the medial head of the gastrocnemius muscle through a branch that ran in close contact with the tibial nerve. Then run caudally to the digital flexor muscles after sending a branch to the lateral insertion of the semitendinosus muscle, and it was divided into two terminal branches, a lateral terminal branch that ran to the dorsal portion of digits V and IV and a medial branch that connected to the lateral distal branch of the tibial nerve (Figures 6 and 7).

### 3.9. Tibial nerve

The tibial nerve provided branches for the triceps surae muscle (gastrocnemius muscle and soleus muscle), the plantaris muscle, the medial digital flexor muscle, the short digital flexor muscle, the popliteal muscle, the caudal tibial muscle, and the long digital flexor muscle of the first digit. Then it was divided into two terminal branches, lateral and medial, that ran caudally to the long digital flexor muscle of the first digit. The lateral branch received the medial terminal branch from the caudal sural cutaneous nerve. It originated the lateral plantar nerve (*N. plantaris lateralis*), which ran medially to the calcaneus, and then it divided into a superficial and a deep branch. The superficial branch provided sensitive innervation to the digits V and IV, while the deep branch provided motor innervation to the short digital flexor muscle of the first digit and the interosseous muscles. The medial branch formed the medial plantar nerve (*N. plantaris medialis*) that provided sensitive innervation for the digits I to IV and their pads (Figures 6 and 7).

### 3.10. Common fibular nerve

The common fibular nerve sent a branch to the biceps femoris muscle and ran along the lateral surface of the thigh under this muscle to the caudal region of the knee. It then sent a branch to the gastrocnemius muscle lateral head. After that, it was divided into the superficial fibular nerve (*N. fibularis superficialis*) and the deep fibular nerve *(N. fibularis profundus)*. The superficial fibular nerve ran along the lateral surface of the crural region, cranially to the tibiotarsal joint, and over the dorsal surface of the foot to finally send sensitive branches for the digits I to IV. The deep fibular nerve provided motor innervation to the cranial muscles from the crural region: fibularis longus, fibularis brevis, long digital extensor, lateral digital extensor, short digital extensor, long extensor of the first digit, and cranial tibial. It also innervated the dorsal muscles of the foot: The short extensor of the first digit and the lumbrical muscles (Figures 6 and 7).

### 3.11. Caudal femoral cutaneous nerves

The caudal femoral cutaneous nerve (*N. cutaneus femoris caudalis*) originated from S1 and ran caudally with the caudal gluteal artery and vein.

### 3.12. Pudendal and ventral coccygeal nerves

**Figure 8.**
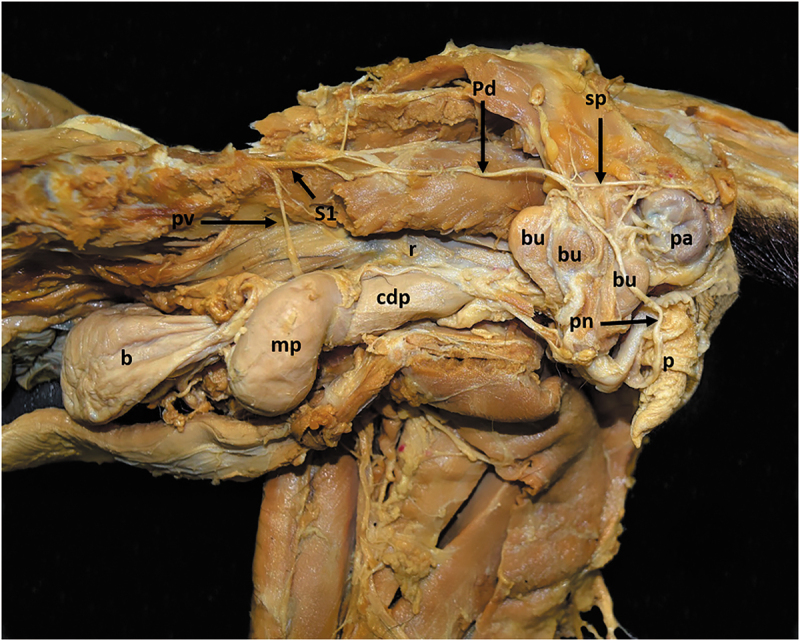
Lateral view of the pelvic cavity to show the visceral innervation. *b*, urinary bladder; *bu*, bulbourethral gland; *cdp*, caudal prostate; *mp*, middle prostate; *p*, penis; *pa*, paranal sinus; *pn*, nerve to the penis; *pd*, pudendal nerve; *pv*, pelvic nerve; *r*, rectum; *S1*, first sacral nerve; *sp*, superficial perineal nerve.

The lumbosacral trunk originated the pudendal nerve (*N. pudendus*) and the ventral coccygeal nerve (*N. caudalis ventralis*). The pudendal nerve was formed by L5 and S1 in the 5VP specimens and from L6 and S1 in the 6VP specimens. It emerged from the major sciatic foramen and ran medially to the ischiotibial branch from the sciatic nerve to finally divide into dorsal and ventral branches. The dorsal branch innervated the paranal sinus and the perineal region, while the ventral branch innervated the penis and the three bulbourethral glands in the male. In the female, this branch ran to the urogenital canal (Figure 8).

The ventral coccygeal nerve ran along the tail, innervating the hypaxial coccygeal musculature. This nerve received a contribution from S2 to form the dorsal coccygeal nerve (*N. caudalis dorsalis*), which innervated the epaxial coccygeal musculature. Distally, these coccygeal nerves received a contribution from the first (Cc1) to the fourth (Cc4) coccygeal nerves.

## 4. Discussion

In this study, we found a variation in the number of lumbar vertebrae (L5 or L6) in the twenty specimens of common opossum dissected while in a more significant sample of specimens of the white-eared opossum (*Didelphis albiventris*) (*n*= 35) was reported a variation in these vertebrae with three different patterns (5VP, 6VP, and 7VP) [13], so it seems that lumbar vertebrae and nerves variability can be extended to the *Didelphis* genus. Other authors have not reported any variation in the number of lumbar vertebrae either in Brazilian common opossum (*D. aurita*) or Virginia opossum (*D. Virginiana*) [14,15]. Further studies with a more significant number of specimens from different geographic areas are desirable to confirm if the common opossum also presents the seven-vertebrae pattern as occurs in the white-eared opossum. Many mammal species have reported a high intraspecific variation in the number of vertebrae. A higher number of lumbar vertebrae has been related to greater flexibility for terrestrial locomotion; however, this is still a polemic topic regarding the developmental biology of the mammalian taxa [18]. A relation between sex and vertebral mutations was shown recently in humans, in which all variations were seen in females [18]. Nevertheless, studies on opossums did not report a relationship between sex and vertebral variations in opossums [13]. In our study, three of twenty opossums had vertebral variation (15%), two males and one female. More studies with a higher number of specimens would be necessary to determine a sex tendency in these variations.

We found three innervation patterns in the 5VP specimens: in 5VPa, L2 formed three nerves (ilioinguinal -with L1-, genitofemoral and caudal femoral cutaneous -only L2-); while in 5VPb and 5VPc, L2 also included the femoral and obturator nerves (with L3). Regarding the origin of the lumbosacral sacral trunk, we can define the 6VP as the common conformation due to its higher frequency, so the 5VP specimens showed a cranial shift in the lumbosacral plexus [19]. The variation in the number of the lumbar vertebrae has been associated with a caudal or a cranial deviation in the lumbar plexus, as we found here in the common opossum. This segmental variation could have clinical implications like lumbar radiculopathies [19]. Thus, it would be fascinating to research this variation in a more significant sample in the common opossum.

In humans also, two vertebral patterns have been reported: 5VP and 6VP, with two nerve distribution patterns in the 5VP [18]. Regarding the 5VP, there are some similarities and differences. The origin of the iliohypogastric nerve (L1) was the same in humans and opossums with 5VPa and 5VPb, while it was different in 5VPc (T13); the ilioinguinal nerve was formed by L1 or L1-L2 in humans, while in opossums it was formed by T13 and L1 in 5VPc, L1 and L2 in 5VPa and 5VPb; the genitofemoral nerve was formed by L2 or L1-L2 in humans, similar to 5VPc (L1-L2), while in the other 5VP it was formed by L2-L3 (5VPa) or L2 (5VPb). The most common spinal nerve contribution to the lateral femoral cutaneous nerve in humans was similar to 5VPa and 5VPc opossums (L2-L3); nevertheless, in humans, this nerve originates less often solely from the L2, similar to 5VPb opossums [20]. The femoral and obturator nerves were formed by L2-L3-L4 in humans, while in opossums, it was formed by L3-L4 (5VPa) and L2-L3 (5VPb and 5VPc). The lumbosacral trunk had a wider origin in humans (L4-L5-S1-S2-S3), while in 5VP opossums it was formed by L4-L5-S1. Both humans and opossums conserve the same lumbosacral trunk origin (between the two last lumbar vertebrae and the first sacral vertebrae), while the other lumbar nerves present a higher variability in their distribution depending on the vertebral count [18]. In 6VP humans, the only similarity to opossums is the origin of the genitofemoral nerve (L2-L3). Differences were seen in the iliohypogastric nerve (L1 or L2 in humans, L1 in opossums), the ilioinguinal nerve (L2 in humans, L1-L2 in opossums), the lateral femoral cutaneous nerve (L2-L3 or L3-L4 in humans, L3 in opossum); the femoral and obturator nerves (L3-L4-L5 in humans, L3-L4 in opossums), and the lumbosacral trunk (L5-L6-S1-S2 in humans, L5-L6-S1 in opossums) [18].

The iliohypogastric nerve origin was similar to domestic herbivores such as ruminants and horses [21] and the Virginia opossum [15]; but differently from non-human primates in which the last thoracic nerve (T12), so-called subcostal nerve, is included in this plexus [22,23]. Previous studies did not describe the contribution of L1 and L2 to the lumbar plexus in the Brazilian common opossum [14]. Our results differed from Chinchillas, which have six lumbar vertebrae, and even so, they presented cranial and caudal iliohypogastric nerves originating from L1 and L2, respectively [22]. According to our results for 6VP, femoral and obturator nerves were formed by L3 and L4 in the Brazilian opossum [14]. However, in the Virginia opossum also received a contribution of L5 [15]. For other mammals with six vertebrae, there is a considerable variation in the origin of these nerves. In chinchilla and Guinea pig emerged from L4 and L5, similar to the 6VP common opossum reported here [22–24]. Otherwise, in rats, the femoral nerve originated from L2, L3, and L4, and the obturator nerve from L3, L4, and L5 [25]. In the case of dogs, pigs, rabbits, and cats that have seven lumbar vertebrae, they also showed different origins for these nerves. For dogs and pigs is L3, L4, L5, and L6; for rabbits is L5, L6, and L7; and for cats is L6 and L7 [23,26–29].

Regarding the lumbosacral trunk, our results agreed with the previous report on the Virginia opossum [15] but not with the study on the Brazilian opossum (L4, L5, and L6, with no contribution from S1) [14]. The branches derived from the lumbosacral trunk were the cranial gluteal nerve, the caudal gluteal nerve, and the sciatic nerve, but neither of these two articles reported the presence of the caudal femoral cutaneous nerve [14,15].

Compared with other species, the lumbosacral plexus in dogs is formed by L5, L6, L7, and S1 [29,30]. It would be necessary to verify if it has similarities in nerve pattern distribution in Didelphis species with 7 lumbar vertebrae [13]. In the case of cats, it also has the participation of L4 (similar to our results in 6VP) and S2 [26]. The lumbosacral plexus in Guinea pigs has a more caudal origin (L6, S1, and S2), and in rabbits, it also has the participation of L5 and S3 [23,24,27]. In chinchillas and pigs, it is formed by L5, L6, S1, and S2 [22,23].

The pudendal nerve was seen emerging from the major sciatic foramen, making it more accessible during surgeries, as reported for the Brazilian common opossum [14]. In contrast with our results, in the Brazilian opossum, the pudendal nerve was formed only by S1 [14], and in the Virginia opossum was reported to be formed by L5, L6, S1, and S2 [15]. The pudendal nerve is formed only by the sacral spinal nerves (S1, S2, and S3) in dogs, while in cats and pigs, S1 does not participate [23,26,29]. In rabbits is formed by S1, S2, S3, and S4, in Guinea pigs by S2, S3, and S4, and in chinchillas only by S1 and S2 [22,24,27]. Interestingly, there is no participation of lumbar spinal nerves in the formation of the pudendal nerve in this species, contrary to the evidence seen in the opossum.

The formation of the lumbosacral plexus in other wild mammals differs from the common opossum. In porcupines (*Hystrix cristata*), it also includes typically the last thoracic spinal nerve in the plexus (T15, L1, L2, L3, L4, S1, and S2), while in this study, only one specimen with 5VPc includes T13 in the plexus. In this species, all the innervation of the pelvic limb comes from L3, L4, S1, and S2 (caudal gluteal nerve, caudal cutaneous femoral nerve, and sciatic nerve), including the sacral segments, unlike the common opossum [31]. The lumbosacral plexus in agouti (*Dasyprocta leporina*) included only the last lumbar spinal nerves and all the sacral spinal nerves (L4, L5, L6, L7, S1, S2, S3, and occasionally, S4). The femoral nerve came out much more caudally than the opossum (L5-L7), the sciatic nerve was formed with the last lumbar spinal nerve, and the three first sacral spinal nerves (L7, S1, S2, and S3), and the pudendal nerve was formed by the interconnections of S2, S3, and S4 [32].

Opossums have many particularities in the muscular anatomy of the pelvic limb. In agreement with our results, it has been described the absence of the tensor fasciae latae muscle, the existence of only one sartorius muscle, and the presence of two origins and three insertions for the semitendinosus muscle in the Brazilian common opossum [14]. In contrast, they did not report the presence of the gluteofemoral muscle. The superficial and deep digital flexors muscles usually found in the posterior tibial musculature of quadrupedal mammals were not recognized; instead, there is a unique long muscle that has its insertion in the intermediate phalange, the long digital flexor muscle, as in humans. This muscle has a short muscle originating in its final tertium that inserts in the distal phalange, the short digital flexor muscle, that replaces the deep digital flexor muscle. Furthermore, they present the plantaris muscle [30,33,34]. The fibular nerve innervated all these muscles.

The opossum foot muscles are similar to the human hand, i.e. the short flexor muscle of the first digit, the long extensor muscle of the first digit, and the short extensor muscle of the first digit [30,33,34]. All these muscles were innervated by the tibial nerve. Opossums’ feet act like a hand because of their opposite thumb. Unfortunately, previous reports on the lumbosacral plexus anatomy of opossum species lack the description of the muscular anatomy of the leg and the feet [14,15].

The common opossum has a high breeding potential in captivity for conservation and commercial purposes [35,36], a good reason to expand the knowledge of the anatomy of this species. The knowledge of the origin and distribution of the lumbosacral plexus is also needed to achieve better medical attention, improving the diagnosis of neuromuscular illnesses and the implementation of local nerve blocks and regional anaesthesia as in other wild mammalian patients [37]. This is particularly important given the variations in the nerve distribution associated with the variable number of lumbar vertebrae.

The analysis of anatomical variations can contribute to obtaining an actual, not idealized or oversimplified, image of anatomical relationships [38]. Considering the particularities reported here and the possibility of easy reproduction and management, this species can be postulated as a good biomodel option [9,14,39].

## 5. Conclusions

There were variations in the lumbosacral plexus anatomy among the specimens of the common opossum due to the variable number of lumbar vertebrae. Also, there were differences between the Brazilian common opossum and the Virginia opossum regarding lumbosacral plexus and pelvic limb muscles anatomy. Further studies for a more thorough description of the musculoskeletal system are required.

## Data Availability

The authors confirm that the data supporting the findings of this study are available within the article.
